# Prognostic and immunological role of cuproptosis-related protein FDX1 in pan-cancer

**DOI:** 10.3389/fgene.2022.962028

**Published:** 2022-08-19

**Authors:** Chen Xiao, Linhui Yang, Liangzi Jin, Weiguo Lin, Faqin Zhang, Shixin Huang, Zhijian Huang

**Affiliations:** ^1^ Department of Gastroenterology, Fuzhou Second Hospital Affiliated to Xiamen University, The School of Clinical Medicine, Fujian Medical University, Fuzhou, China; ^2^ The Graduate School of Fujian Medical University, Fuzhou, China; ^3^ Institute of Medical Biology, Chinese Academy of Medical Sciences and Peking Union Medical College, Kunming, China; ^4^ Department of Ultrasound, Fujian Medical University Cancer Hospital, Fujian Cancer Hospital, Fuzhou, China

**Keywords:** copperoptosis, pan-cancer, prognosis, biomarkers, immunological

## Abstract

**Background:** Cancer is the second cause of death worldwide. Copperoptosis is a new mode of regulated cell death and is strongly associated with metabolic pathways. FDX1 is a key gene that promotes copperoptosis, and its impact on tumor pathogenesis and tumor immune response is indistinct and needs further exploration.

**Methods:** Data was mined from the Cancer Genome Atlas database, the Broad Institute Cancer Cell Line Encyclopedia database, and the International Cancer Genome Consortium. Survival analyses included the Kaplan–Meier method for calculating the cumulative incidence of survival events and the log-rank method for comparing survival curves between groups. Immune cell infiltration levels were calculated using the Spearman correlation test and correlated with FDX1 expression to assess significance. More correlation analyses between FDX1 expression and mutational markers, such as tumor mutational burden (TMB) and microsatellite instability (MSI), were also examined *via* Spearman assay to explore the relation between FDX1 expression and the sensitivity of common antitumor drugs.

**Results:** FDX1 expression was downregulated in most kinds of cancers, and this high expression indicated better overall survival and death-specific survival. For several cancer types, FDX1 expression had a positive correlation with immune cell infiltration, and FDX1 also had a positive correlation with TMB and MSI in some cancer types, linking its expression to the assessment of possible treatment responses.

**Conclusion:** The correlations between FDX1 expression and cancer in varioustissues, including clear links to cancer survival and prognosis, make FDX1 aninteresting biomarker and potential therapeutic target for cancer surveillance and futureresearch.

## Introduction

Cancer is the second cause of death worldwide. In 2020, approximately 19.3 million new cancer cases were found worldwide. Female breast cancer has become the commonest cancer diagnosed with approximately 2.3 million new cases (11.7%) exceeding lung cancer (11.4%), colorectal cancer (10.0%), prostate cancer (7.3%), and gastric cancer (5.6%) (Liu et al., 2021; Sung et al., 2021). Cancer is driven by genetic change, and the occurrence and development of cancer can be divided into three stages: transformation and growth of carcinogenic factors, promotion, and development of carcinogenesis. This is a multifactor, multistep complex process. Metabolism is significant in carcinogenesis, and recently, metabolism-targeted therapy has become an important part of tumor therapy. As tumorigenesis is complex, the conduction of a pan-cancer expression analysis of any gene of interest and the assessment of its correlation with clinical prognosis and potential molecular mechanisms are important. The publicly funded TCGA project contains functional genomics datasets of different tumors so pan-cancer analyses can be conducted (Tomczak et al., 2015; Blum et al., 2018; He et al., 2020a; He et al., 2020b; Zhao et al., 2021; Yang et al., 2022).

FDX1, also called adrenodoxin or hepatoredoxin, is a subunit of the augmin complex. The FDX1 gene is a small ferrithionein that transfers electrons from NADPH to mitochondrial cytochrome P450 *via* ferredoxin reductase, involved in the metabolism of steroids, vitamin D, and bile acids (Sheftel et al., 2010; Strushkevich et al., 2011). Diseases associated with FDX1 include cerebrotendinous xanthomatosis and xanthomatosis. The latest research shows that the FDX1 gene is a recently discovered important gene associated with copperoptosis (Tsvetkov et al., 2022). FDX1 positively regulates a specific metabolic pathway of copperoptosis, and FDX1 and Protein lipoylation are key regulators of copper ion carrier-induced cell death. FDX1 is associated with protein thioctanoylation, and FDX1 knockout results in loss of protein thioctanoylation. Protein thioctanoylation is a highly conserved posttranslational modification of lysine that occurs primarily on four enzymes that regulate the tricarboxylic acid cycle. Copper ions promote cell death by directly binding to thioctanoylated tricarboxylic acid cycle-related enzymes, and the knockout of FDX1 can save cell copperoptosis.

Copperoptosis is a new mode of regulating cell death (Tang et al., 2022). Copper ions are involved in cell death such as iron ions (Wang et al., 2022). Inhibiting mitochondrial respiration through drugs may be a strategy to fight diseases. In addition, some cancers express a large amount of thioctanoylated mitochondrial proteins, and with high respiration, the use of copper ion metal carriers to kill cancer cells may become a new method of treating cancer. FDX1 gene is a key gene that promotes copperoptosis, so the study of FDX1 is significant for tumorigenesis, progression, tumor prognosis, tumor treatment, and many other aspects in practice (Zhang et al., 2021). Here, bioinformatics analyses were conducted to evaluate different FDX1 expressions in tissues and their possible link with cancer. Its expression level was evidently associated with survival, immune cell function, and tumor mutation status. FDX1 can be used as a new prognostic marker for various malignancies and an indicator of cancer immunotherapy response.

## Materials and methods

### Data collection and processing

Pan-cancer sequencing data from the Cancer Genome Atlas (TCGA) database and the Broad Institute Cancer Cell Line Encyclopedia (CCLE) database (Illumina platform) and data related to hepatocellular carcinoma (LIHC) from the International Cancer Genome Consortium (ICGC)) databases were drawn through their portal for analysis (Hudson et al., 2010; Tomczak et al., 2015). The entire data set was screened, and missing and duplicate results were removed and converted by log2 (TPM + 1), using the rma function in the R package (R studio version: 1.2.1335, R version: 3.6.1). Relating information of clinic was also drawn through the portal, including the patient’s age, gender, tumor stage, and clinical stage. In addition, the information that can only be downloaded from the TCGA database were tumor mutation load (TMB) and microsatellite instability (MSI). The calculation of TMB followed the total mutation incidence per million base pairs, and the calculation of MSI was from the amount of insertion or deletion events in a repeating genetic sequence. Data analysis was conducted using the Sangerbox tools (http://sangerbox.com/).

### Cox regression analysis and survival analysis

In the ICGC and TCGA, Cox regression analysis was conducted to find out if FDX1 expression correlated with overall survival (OS) and disease-specific survival (DSS) for patients with different cancer types. Using the Kaplan–Meier method, the patients were grouped into high and low FDX1 expressions according to the optimal separation method, and the survival curve of patients with various cancer types was constructed. The analysis of specificity and time-dependent sensitivity of survival was conducted by deploying survival ROC and survival in R packages (rdocumentation.org/packages/survival). The difference between curves was checked via a log-rank test, and *p* values of less than 0.05 were regarded as important.

### Immune cell infiltration and enrichment

Tumor Immune Estimation Resource (TIMER) (https://cistrome.shinyapps.io/timer/) is a computational network tool based on a database for immune cell infiltration that supplies infiltration scores for six common immune cell types, including B cells, CD4 + T cells, CD8 + T cells, macrophages, neutrophils, and dendritic cells (Li et al., 2016; Yang et al., 2017; Tavasolian et al., 2020; Hwu et al., 2021). The calculation of immune cell infiltration scores for pan-cancer data in the TCGA database was performed using TIMER and archived online. Here, correlation with FDX1 expression was tested with downloaded penetration data.

### Correlation analysis of FDX1 expression in tumor microenvironment

The immune tumor microenvironment (TME) is a tumor cell–developing and –surviving microenvironment. It involves different elements surrounding tumor cells, stromal cells, etc. (Arneth, 2019; Liu et al., 2022a). The stromal and immune cell quantity in the tumor microenvironment affects the development and growth of cancer cells. The R package “ESTIMATE” is used to calculate StromalScore, ImmuneScore, and ESTIMATEScore, which is the sum of ImmuneScore and StromalScore (Yoshihara et al., 2013; Lv et al., 2021). Then, Spearman correlation analysis in R was used to analyze the association between FDX1 and stromal and immune scores.

### Correlation analysis between FDX1 expression and immune infiltrating cell expression

TIMER is a database providing a platform for tumor immunoinfiltration analysis (Yang et al., 2017). In general calculate the infiltration scores of six types of immune infiltrating cells: CD4 T cells, B cells, CD8 T cells, neutrophils, dendritic cells, and macrophages. The “gene” module in TIMER was used for the analysis of the correlation between FDX1 expression in the TCGA database and levels of immune infiltration across multiple cancer types.

### Drug susceptibility analysis

A total of 60 cancer cells listed by the National Cancer Institute (NCI) Cancer Research Center are the basis of the CellMiner database. The NCI-60 cell line is the most popular cancer cell sample group for anticancer drug detection recently. Here, NCI-60 drug sensitivity data and RNA-seq gene expression data were downloaded, and the relation between genes and the sensitivity of common antitumor drugs was explored through correlation analysis.

### Statistics

Correlations between FDX1 expression and target targets were assessed using Spearman correlation tests, including immune cell infiltration scores (as the description in the previous section for the six immune cell types), TMB, MSI, and mismatch repair (MMR) genes. According to whether the samples were paired, FDX1 expression levels were compared between groups or between tumors and normal tissue using paired *t*-test or *t*-test. *p* values below 0.05 are regarded as evident. All charts are generated from the R package of ggplot2 and forestplot.

## Results

### Expression levels of FDX1 in various normal and cancerous tissues

**FIGURE 1:**
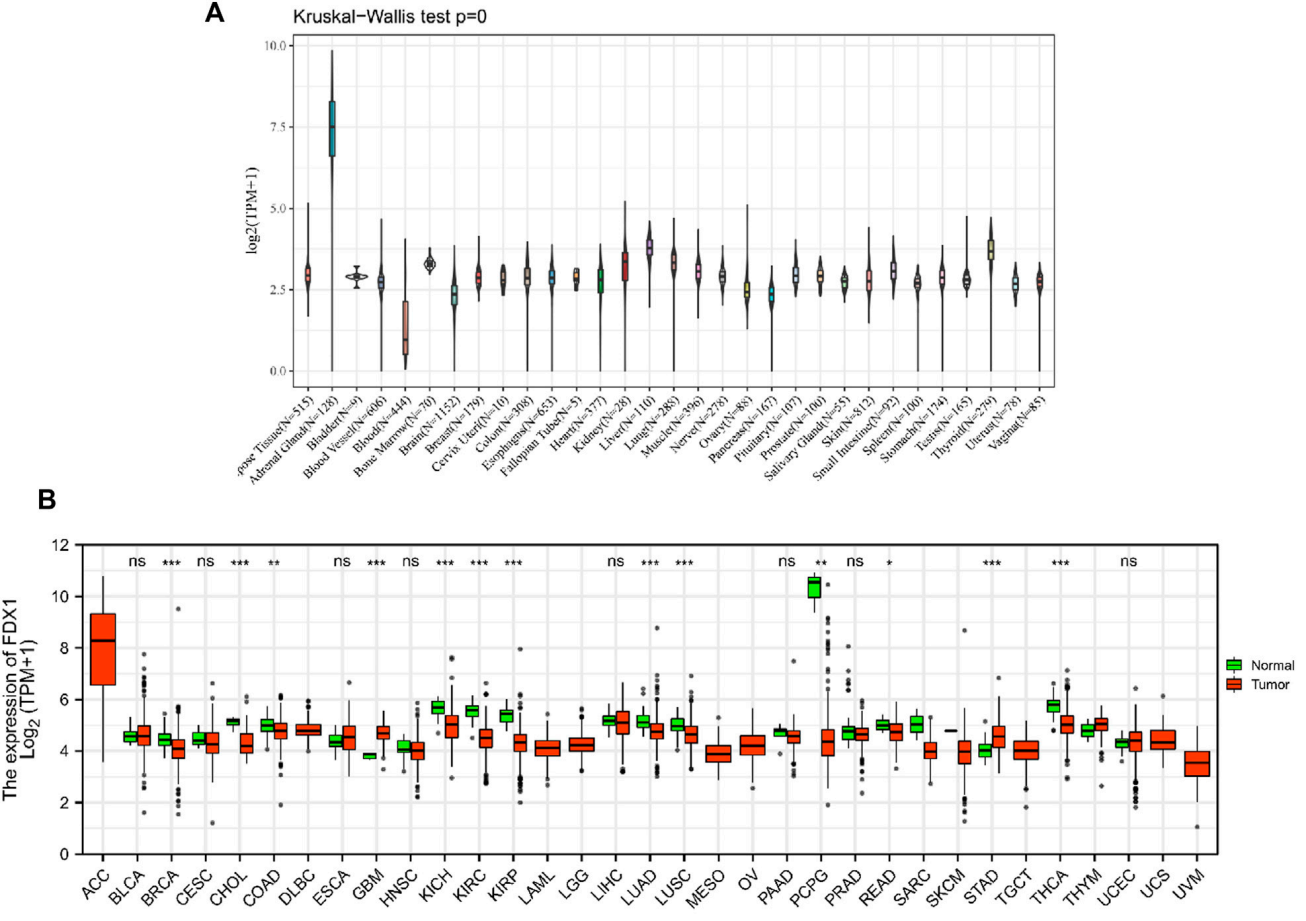
mRNA expression levels of FDX1 from different tissue sources and tumors. **(A)** normal mRNA expression levels of FDX1 in various tissues from the GTEx database. **(B)** differences in FDX1 mRNA expression between tumor and peritumoral samples from the Cancer Genome Atlas database. Abbreviations: BLCA, bladder urothelial carcinoma; CESC, cervical and cervical cancer; CHOL, cholangiocarcinoma; COAD, colon adenocarcinoma; DLBC, lymphoid tumor diffuse large B-cell lymphoma; ESCA, esophageal cancer; HNSC, head and neck squamous cell carcinoma; KIRP, renal papillary cell carcinoma; LUAD, lung adenocarcinoma; LUSC, lung squamous cell carcinoma; PAAD, pancreatic cancer; PCPG, pheochromocytoma and paraneurysm; PRAD, prostate adenocarcinoma; reading, rectal adenocarcinoma; SARC, sarcoma; STAD, gastric adenocarcinoma; STES, gastric and esophageal cancer; TGCT, testicular germ cell tumor; THCA, thyroid cancer; THYM, thymoma; UCEC, endometrial cancer of the uterus; UCS, uterine carcinosarcoma.

With the data of GTEx databases from different tissues in healthy individuals, it was determined that mRNA expression levels of FDX1 were similar in all tissues (Figure 1A), except for the adrenal gland. As an actively differentiated tissue, the higher expression levels of the adrenal gland were not unexpected. Further comparison of relatively normal tissues and respective tumors showed that FDX1 was lowly expressed in most tumors, except for GBM and STAD, showing that the opposite result was significant. Based on TCGA data, 13 of 33 cancer types (BRCA, CHOL, COAD, GBM, KICH, KIRC, KIRP, LUAD, LUSC, PCPG, READ, STAD, and THCA) showed significant differences in expression (Figure 1B).

### Analysis of the relationship between FDX1 expression level and prognosis

**FIGURE 2:**
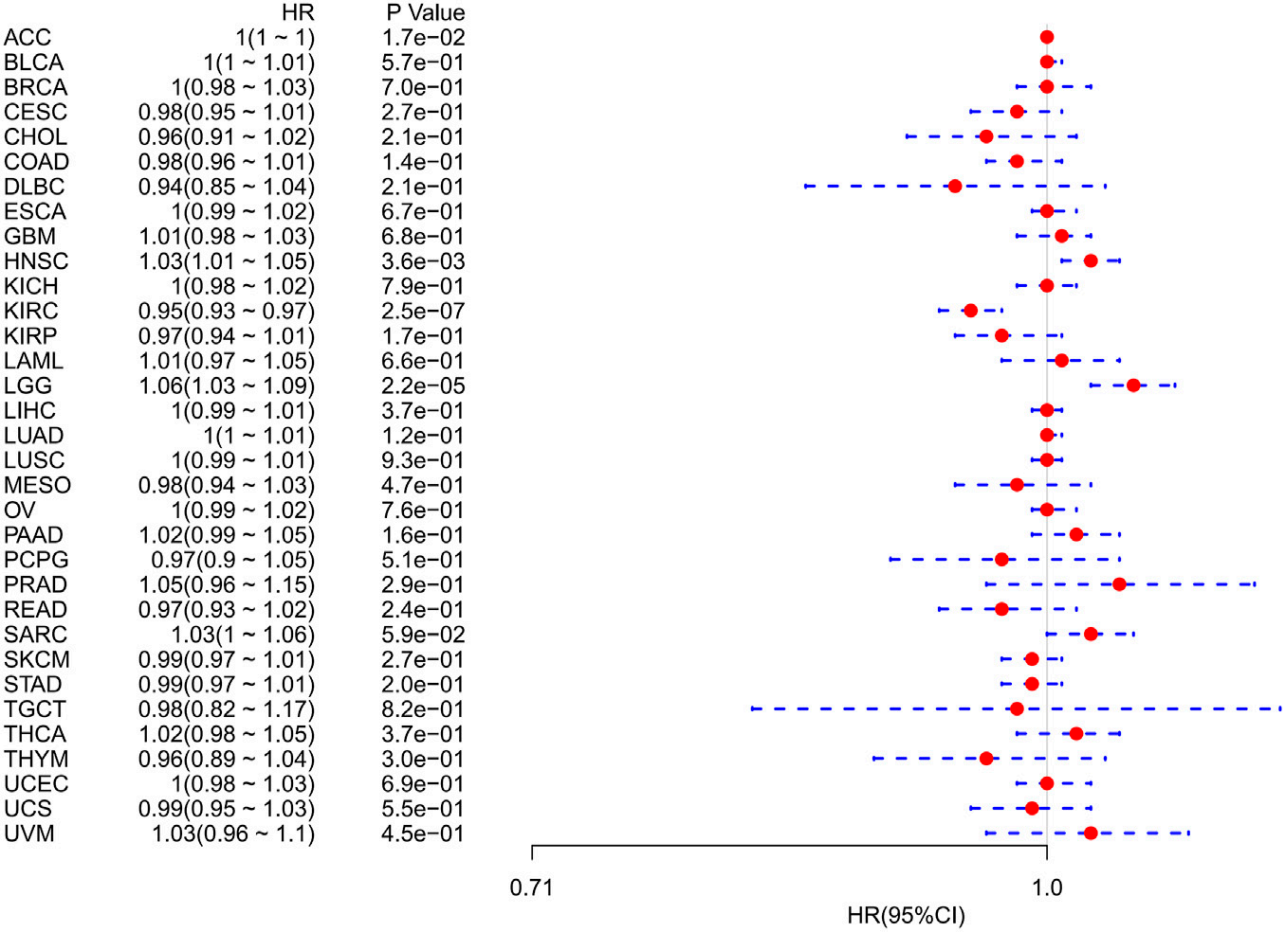
Association of FDX1 mRNA expression levels with overall survival in multiple tumors from the Cancer Genome Atlas database. Cox regression analysis, *p* < 0.05 was evident.

**FIGURE 3:**
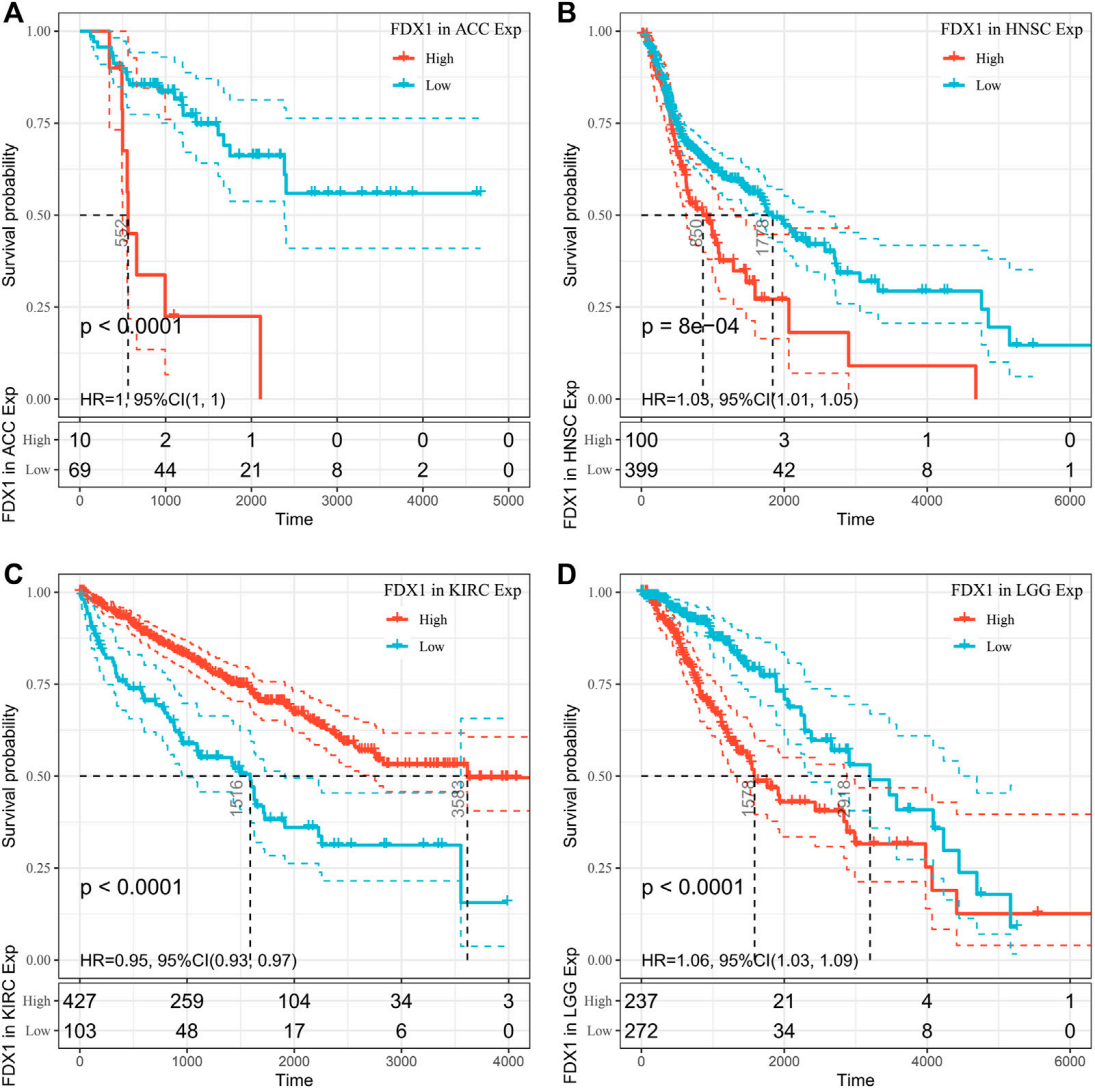
Overall survival (OS) difference of high and low FDX1 mRNA expression groups in significantly prognostically relevant tumors from the Cancer Genome Atlas database (by median expression dichotomy). **(A)** OS difference of ACC groups. **(B)** OS difference between HNSC groups. **(C)** OS difference between KIRC groups. **(D)** OS difference of LGG groups. *p* < 0.05 was regarded significant, with a dashed line of 95% CI.

Using univariate Cox regression analysis, we used data from the TCGA database to assess the correlation between the respective expression levels of FDX1 and OS in various cancers. The hazard ratios of FDX1 to ACC, HNSC, KIRC, and LGG were significant, with FDX1 having the highest risk in LGG, and being a tumor suppressor factor in KIRC (Figure 2). The survival analysis below, using patient data using the median expression value dichotomy for each cancer type (Figure 3), showed that survival differences were significant across OS-related cancer categories and that patients with high FDX1 expression had a better prognosis in some cancers.

**FIGURE 4:**
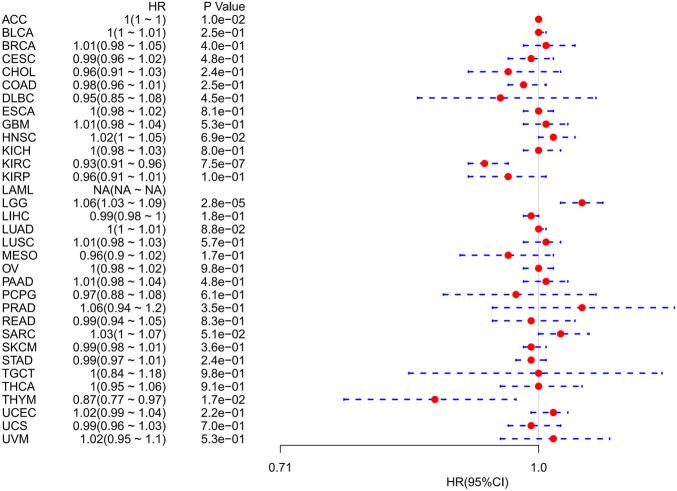
Correlation between FDX1 mRNA expression levels and disease-specific survival in multiple tumors from the Cancer Genome Atlas database. Cox regression analysis, *p* < 0.05 was evident.

**FIGURE 5:**
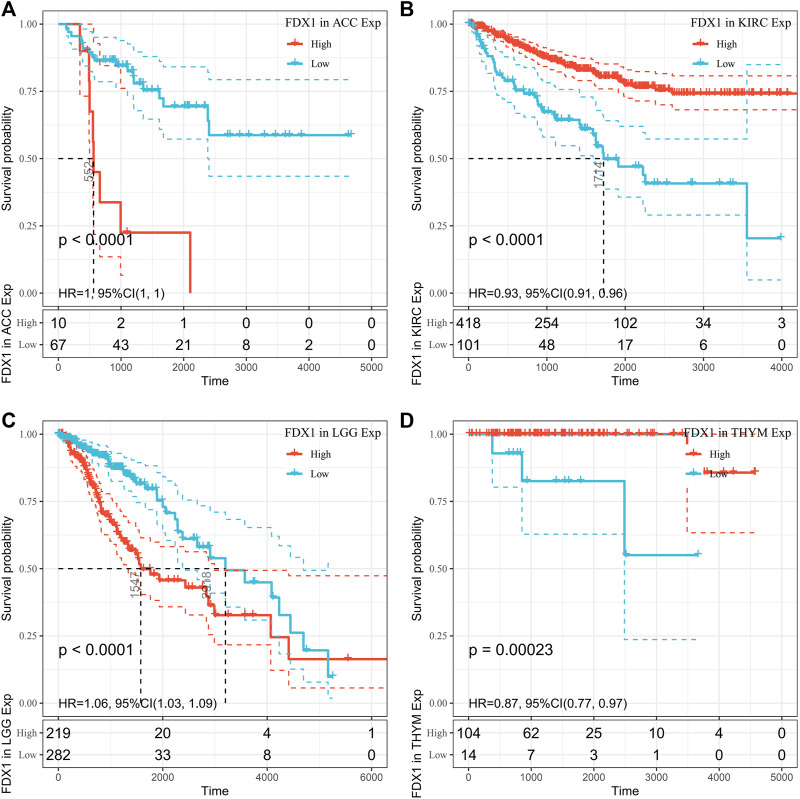
Disease-specific survival (DSS) difference between high and low FDX1 mRNA expression groups in significantly prognostically relevant tumors from the Cancer Genome Atlas database (by median expression dichotomy). **(A)** DSS differences between adrenal cortical carcinoma groups. **(B)** DSS differences between KIRC groups. **(C)** DSS differences between groups in LGG. **(D)** DSS differences between thymoma groups. *p* < 0.05 was considered a significant, 95% CI dashed line.

However, OS may be influenced by noncancer-related deaths during follow-up. Therefore, the data on the correlation between DSS and FDX1 expression in various cancers were reanalyzed (Figure 4). The Cox regression analysis results were similar to those related to OS. Differences included the determination of a significant risk effect on THYM (except for the four cancers mentioned earlier, HNSCs were excluded for *p* greater than 0.05) and the calculation inability of the hazard ratio for FDX1 in LAML due to deficient relevant data. Cancer types with high FDX1 expression (KIRC, THYM) showed a favorable prognosis compared with the low expression group as learned from the later survival analysis (Figure 5).

### Correlation analysis of FDX1 with tumor microenvironment, immune infiltrating cells and immune-related cells in some immune pathways

**FIGURE 6:**
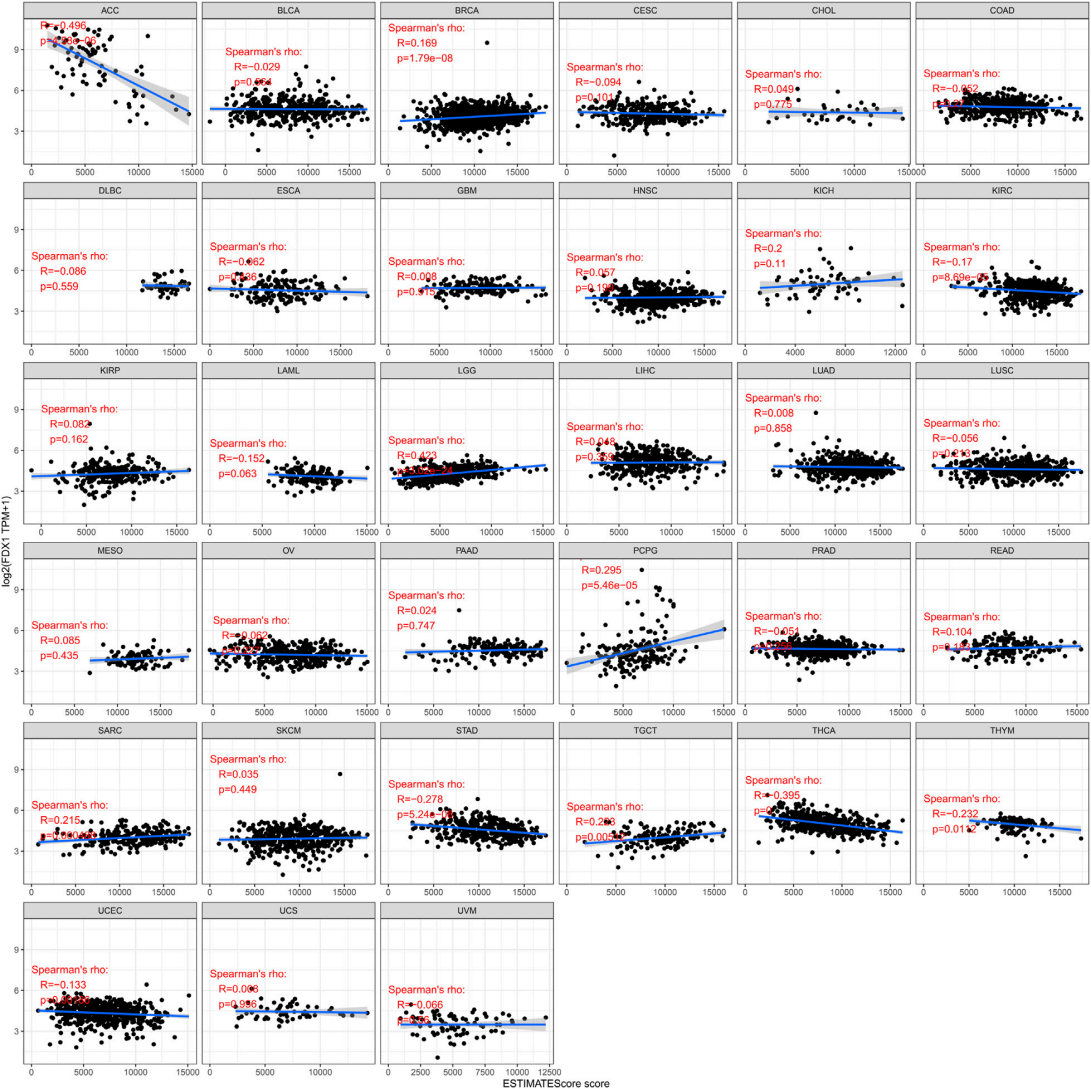
Correlation of FDX1 expression with ESTIMATEScore score in pan-cancer.

The relation between FDX1 and immune and stromal scores was measured. We then visualize the remarkable results (Figure 6). As shown, immune scores in 11 of the 33 cancers were significantly associated with FDX1 expression, and ESTIMATEScore scores in 14 of the 33 cancers were significantly associated with FDX1. BRCA (*r* = 0.169, *p* < 0.05), LGG (*r* = 0.423, *p* < 0.05), PCPG (*R* = 0.295, *p* < 0.05), SARC (*r* = 0.215, p r = 0.223, *p* < 0.05) show a positively correlated. The highest correlation coefficient is LGG. In ACC (*r* = −0.496, *p* < 0.05), KIRC (*r* = −0.17, *p* < 0.05), THCA (*r* = −0.395, *p* < 0.05), THYM (*r* = −0.232, *p* < 0.05), UCEC (*r* = −0.133, *p* < 0.05) show a negative correlation. The highest correlation coefficient is ACC (Stepien et al., 2017). The lower the expression of FDX1, the higher the purity of tumor cells in some kinds of cancers.

The correlation between FDX1 and immunoinfiltrating cells in 33 kinds of cancers in the TIMER database was investigated.

### FDX1 may modulate the tumor immune microenvironment by affecting immune infiltration in various cancer types

**FIGURE 7:**
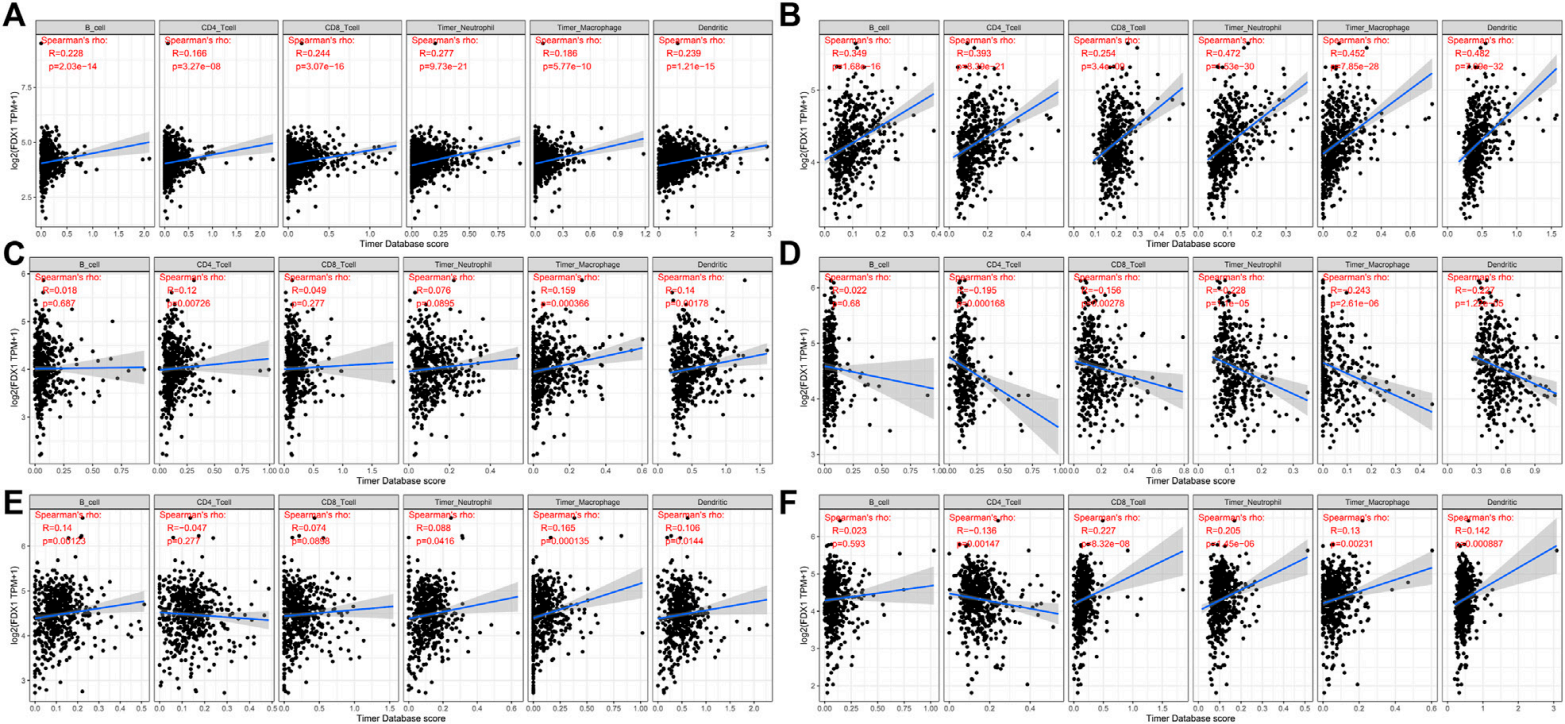
Correlation of six immune cell (B cells, CD4 + T cells, CD8 + T cells, neutrophils, macrophages, and dendritic cells) infiltration scores with FDX1 mRNA expression in six kinds of cancer [**(A)**: BRCA, **(B)**: IGG, **(C)**: HNSC, **(D)**: STAD, **(E)**: KIRC, and **(F)**: UCEC]. Spearman correlation test, *p* < 0.05 was significant.

FDX1 expression and levels of immune cell infiltration in each cancer type were correlated to assess whether this pathway affects the tumor’s immune microenvironment. Several tumors were found by using six immune cell types (B cells, CD4 + T cells, CD8 + T cells, neutrophils, macrophages, and dendritic cells) available in the TIMER database, derived from TCGA. There is indeed a significant correlation. We picked FDX1 with BRCA, HNSC, KIRC, LGG, STAD, and UCEC. Their corresponding linear regression plots showed that in most tumors, high FDX1 expression was correlated with potentially increased levels of immune cell infiltration. In particular, in STAD, FDX1 expression corresponded negatively with immune cell infiltration levels (Figure 7).

### Correlation of FDX1 expression with certain immune checkpoint genes expression in some cancers

Several genes were now closely correlated to and considered checkpoint components in the immune response. The mRNA sequence database allowed assessing whether a link between FDX1 expression and the expression of such checkpoint genes exists. Correlation analysis of FDX1 with checkpoint gene expression found a high correlation (*p* < 0.05) with tumor necrosis factor (TNF)–related immune genes (TNFRSF14, 15, 25) and CTLA4, PDCD1, CD274, NRP1, and VTCN1 in some kinds of cancers.

**FIGURE 8:**
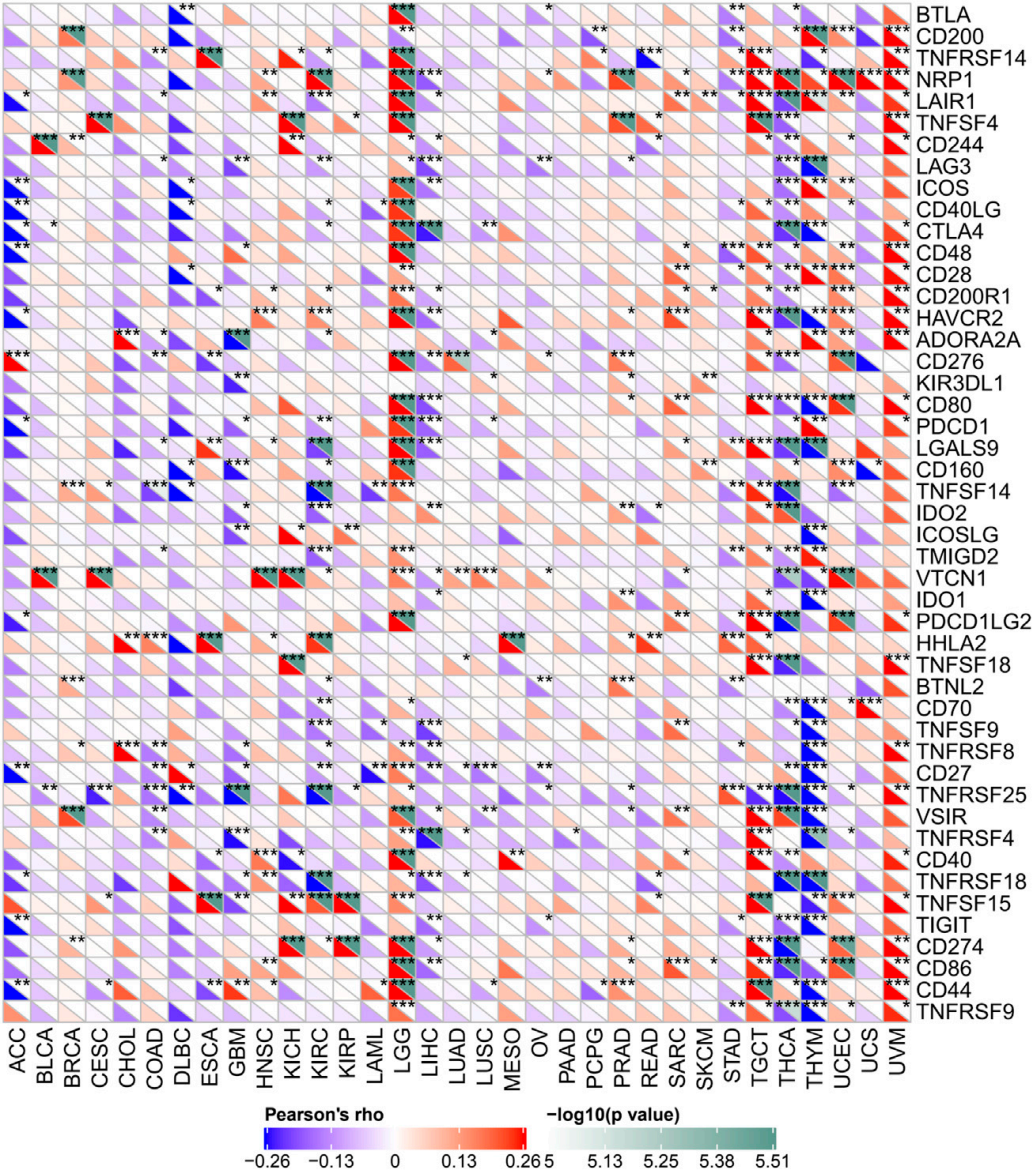
Relation between FDX1 mRNA expression levels and mRNA expression at recognized immune checkpoints in multiple tumors from the Cancer Genome Atlas database. The lower triangle refers to the coefficients calculated by Pearson’s correlation test, and the upper triangle represents the *p*-value converted by log10. *p < 0.05, **p < 0.01, ***p < 0.001.

Moreover, in LGG and TGCT, THCA and THYM, important coexpressions of FDX1 with more immune checkpoint genes, were examined. The results, especially for LGG and TGCT, suggest that FDX1 modulates tumor immune responses by modulating immune checkpoint activity. In addition, in THCA and THYM, FDX1 expression was inversely related with most immune checkpoint molecules but not to a significant extent for some of them (Figure 8).

### FDX1 is related to tumor mutational burden and microsatellite instability in some cancers

**FIGURE 9:**
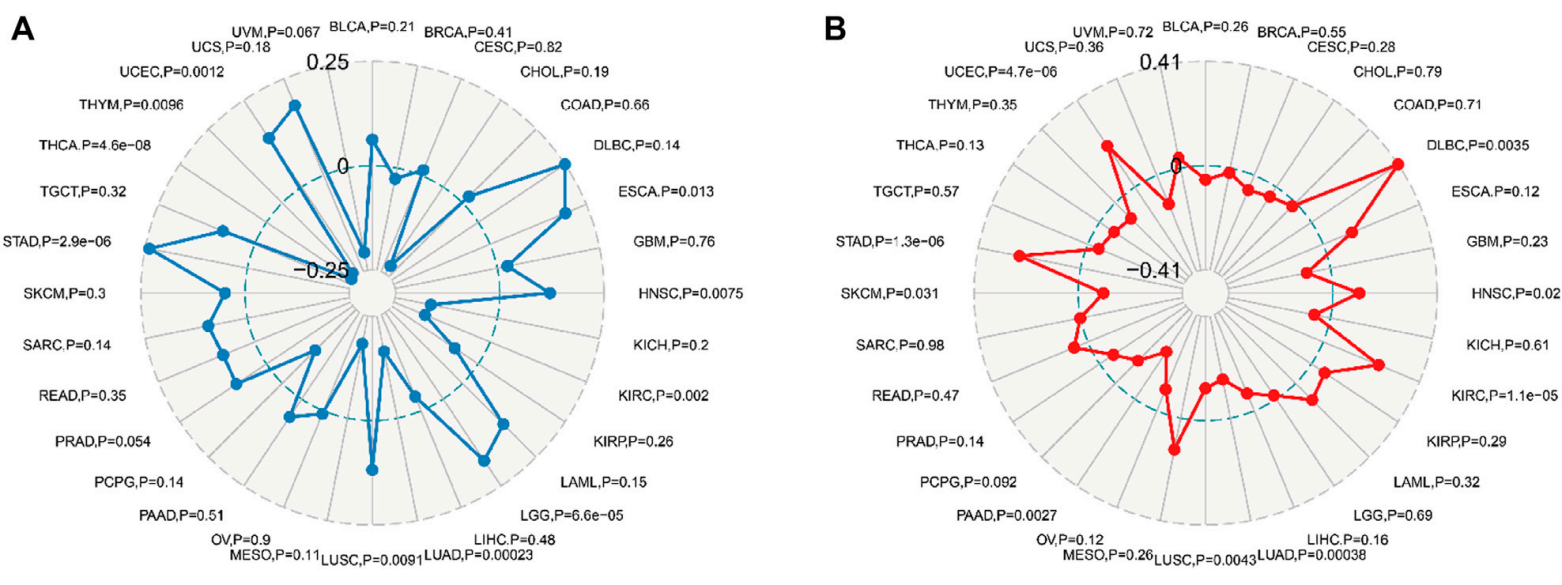
Relationship between tumor mutational burden (TMB), microsatellite instability (MSI), and FDX1 mRNA expression levels in different tumors in the Cancer Genome Atlas database. TMB was calculated by the total mutation incidence per million base pairs in each tumor, and MSI was calculated by the total incidence of deletions or insertions per million base pairs of repeats. **(A)** correlation between TMB and FDX1 expression. **(B)** correlation between MSI and FDX1 expression. Spearman correlation test, *p* < 0.05 was evident.

TMB and MSI are potent prognostic biomarkers and indicators of immunotherapy response in a variety of tumors. Their respective relationships to FDX1 expression in various cancers were examined to investigate the link between FDX1 activity and mutations in specific cancer types. The relation between FDX1 expression and TMB was significant (*p* < 0.05), and data were available for 10 of 32 cancer types (ESCA, HNSC, KIRC, LGG, LUAD, LUSC, STAD, THCA, THYM, and UCEC), among which ESCA, LGG, and STAD coefficients were the highest, whereas KIRC, LUAD, and THCA coefficients were the lowest (Figure 9A). Coefficient values showed that FDX1 expression was positively associated with high mutation status in ESCA, LGG, and STAD but positively correlated to low mutation status in KIRC, LUAD, and THCA (especially THCA).

The relation between FDX1 expression and MSI was examined in 32 cancer types, and the correlation was statistically significant (*p* < 0.05) in nine cancer types (DLBC, HNSC, KIRC, LUAD, LUSC, PAAD, SKCM, STAD, and UCED) (Figure 9B). Among these cancer types, SKCM, PAAD, LUSC, LUAD, FDX1 expression, and MSI had a significant negative correlation, and the PAAD coefficient was the highest; conversely, in DLBC, HNSC, KIRC, STAD, and UCEC, FDX1 expression was positively correlated to MSI, and the DLBC coefficient was the highest. In particular, the STAD cohort had relatively high absolute coefficients associated with either TMB or MSI compared with other kinds of cancers; however, all the quantity of cancer categories showing evident associations with these mutational indicators was lower.

### Pan-cancer expression and drug sensitivity

**FIGURE 10:**
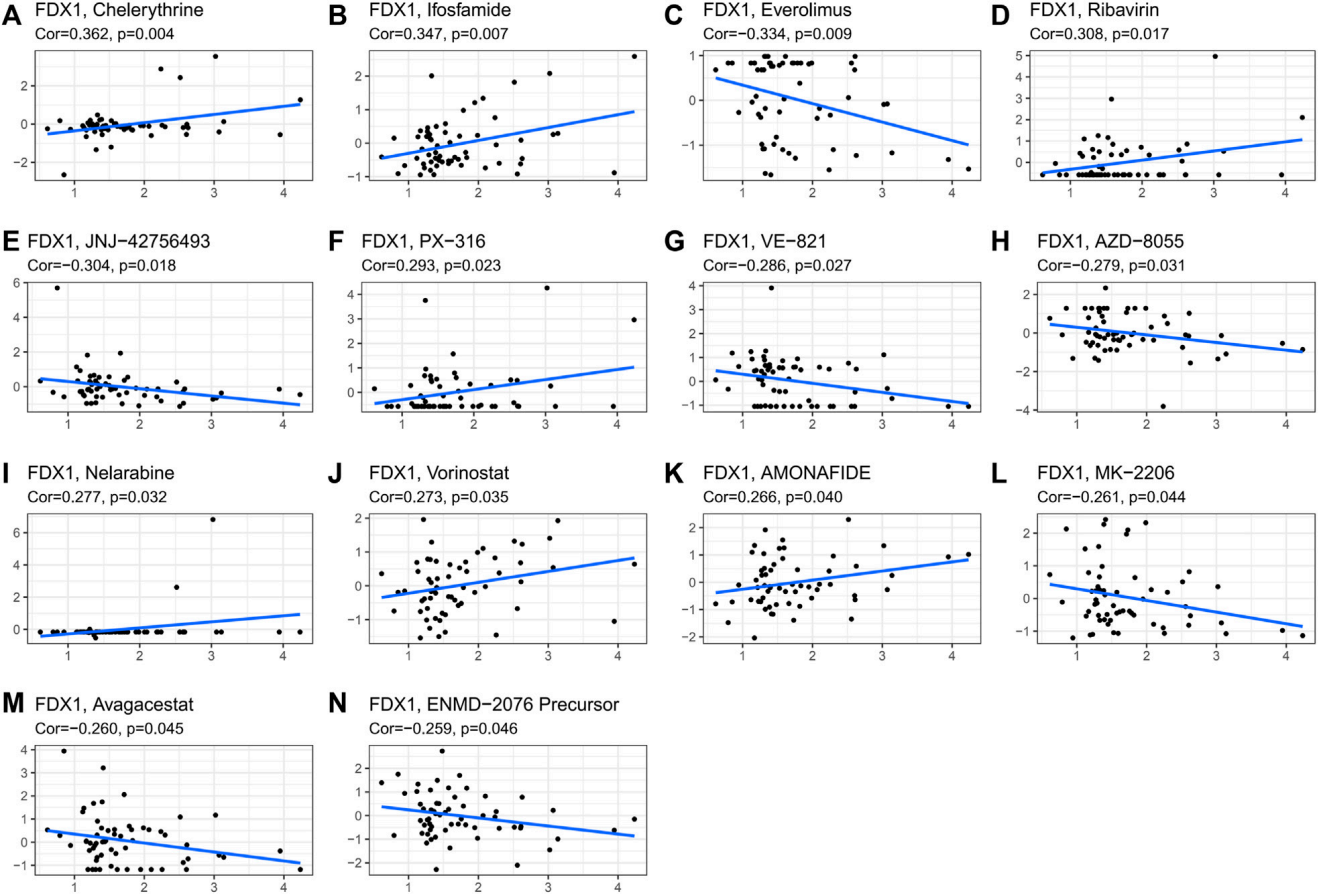
Correlation graph with drug IC50. The correlation graph of gene and drug IC50 and the slope of the straight line are the correlation coefficients between gene and drug. [**(A)**: chelerythrine, **(B)**: ifosfamide, **(C)**: everolimus, **(D)**: ribavirin, **(E)**: JNJ-42756493, **(F)**: PX-316, **(G)**: VE-821, **(H)**: AZD-8055, **(I)**: nelarabine, **(J)**: vorinostat, **(K)**: AMONAFIDE, **(L)**: MK-2206, **(M)**: avagacestat, and **(N)**: ENMD-2076 precursor].

The CellMiner database was used to study the sensitivity of the FDX1 gene to common antitumor drugs and further calculate the correlation between gene expression and the drug IC50. Studies have shown that high expression of the FDX1 gene is associated with resistance to multiple antitumor drugs (Figure 10). Among them, FDX1 was negatively correlated with everolimus, JNJ-42756493, VE-821, AZD-8055, FDX1, MK-2206, avagacestat, and ENMD-2076 precursor and positively correlated with chelerythrine, ifosfamide, ribavirin, PX-316, nelarabine, vorinostat, and amonafide.

## Discussion

Previous studies have shown that FDX1 is necessary for the synthesis of kinds of steroid hormones (Sheftel et al., 2010; Strushkevich et al., 2011). Mitochondrial cytochrome P450 is involved in reducing steroid production (Sheftel et al., 2010; Strushkevich et al., 2011). Its associated pathways include metabolic and inflammatory pathways. It has been reported that FDX1 can enhance the copper-dependent cell death induced by elesclomol and can offer new ideas to improve the efficacy of several cancer-targeted drugs. In addition, FDX1 can augment the copper-dependent cell death induced by elesclomol and can offer a new idea to promote the effect of some cancer-targeting agents (Tsvetkov et al., 2019). Current studies have shown that FDX1 is a key gene that promotes copperoptosis. Copperoptosis is a newly discovered mode of regulatory cell death, varying from other regulatory cell death characteristics such as pyroptosis, ferroptosis, and apoptosis. The relationship between copperoptosis and tumors: It has been found that patients with different cancers (such as breast cancer, thyroid cancer, cervical cancer, ovarian cancer, lung cancer, pancreatic cancer, prostate cancer, breast cancer, oral cancer, and bladder cancer) have serum and tumor tissue copper content that is significantly changed (Baltaci et al., 2017) (Stepien et al., 2017). Copper also promotes angiogenesis, which is critical for tumor progression and metastasis (Ruiz et al., 2021; Ge et al., 2022). Overloaded copper can also lead to cell death. Since copper is important for the occurrence and progression of cancer, it is of great biological significance to study genes related to copperoptosis (Shanbhag et al., 2021).

Our findings suggest that FDX1 is widely expressed in different normal tissues and is relatively high in the adrenal gland. When tumors were compared with corresponding normal tissues, FDX1 expression was reduced in various cancers, and this high expression was associated with better OS and death-specific survival in some cancer types, such as KIPC.

Tumor cells can change the nature of the microenvironment, which in turn can influence tumor growth and spread. Immune cells and stromal cells in the tumor microenvironment can affect cancer prognosis and patient survival outcomes (Ren et al., 2018). TME is strongly associated with tumor occurrence and metastasis (Spill et al., 2016; Liu et al., 2022b; Ye et al., 2022). Previous studies have shown that cytokines in the tumor microenvironment regulate immune function and ultimately suppress immune responses, leading to tumor progression (Hinshaw and Shevde, 2019). Tumor-infiltrating lymphocytes (TILs) in TME have been shown to be independent predictors of prognosis and immunotherapy efficacy in cancer patients (Ohtani, 2007; Azimi et al., 2012). Both immune cells and stromal cells are contained in the tumor environment, and they can determine the role of TME to some extent. Besides, it is reported that immune cells are evidently related to tumorigenesis and development in many researches. Thus, components analyzed in TME contribute to the development of targeted drugs for tumor immunotherapy. We found that FDX1 expression was apparently positively associated with immune cell infiltration in most tumors, whereas in STAD, FDX1 expression was negatively correlated with immune infiltration. In particular, FDX1 expression is also associated with the statistically significant presence of some specific immune checkpoint genes in multiple tumors, such as CTLA4, PDCD1, CD274, NRP1, and VTCN1. Upregulation of this checkpoint gene is associated with escape mechanisms in the immune microenvironment, which suggested that FDX1 plays a role in different immunomodulatory effects in various cancer types.

Our study also found that FDX1 was positively correlated to TME immune, stromal, and ESTIMATE scores in most human cancer types. In addition, the association of FDX1 with TMB and MSI also proves that FDX1 is strongly associated with TME in human cancer. Previous studies had demonstrated that TMB and MSI were markers of drug response in patients, especially those targeting immune checkpoint inhibitors, such as CTLA4 or PD-1/PD-L1 inhibitors (Overman et al., 2017; Mariathasan et al., 2018; Kwon et al., 2020; Shim et al., 2020). In gastric cancer, an analysis of the MAGIC study showed that MSI-H patients might have worse OS after perioperative treatment. Patients with MSI-H/dMMR (deficiency of MMR, dMMR) had many tumor mutations and a wide range of immunogenicity, so they responded well to PD-1/PD-L1 inhibitors. Here, both TMB and MSI of STAD were positively correlated to FDX1 expression, which would support our claim that, of course, FDX1 might be indicating potential drug response (and MSI) well in STAD.

Using the CellMiner study, the result that high expression of the FDX1 gene was associated with resistance to multiple antitumor drugs was obtained. Among them, FDX1 was negatively correlated with everolimus, JNJ-42756493, VE-821, AZD-8055, FDX1, MK-2206, avagacestat, and ENMD-2076 precursor and positively correlated with chelerythrine, ifosfamide, ribavirin, PX-316, nelarabine, vorinostat, and amondafide. We found that FDX12 could serve as a potential resistance target that could predict tumor cell susceptibility to chemotherapy drugs.

Although our study provides useful indications that FDX1 is involved in tumorigenesis and regulation of the immune environment of tumor cells, it does have some limitations. First, as a pure bioinformatics analysis, it relies entirely on information available in open access databases and has not been confirmed experimentally. Here, the assessment of FDX1 expression was based solely on the mRNA levels reported in the aforementioned database, although this cannot show functional protein levels. For instance, protein activity in normal or cancer cells may be affected by posttranscriptional modifications and/or regulatory proteolysis. Future studies will focus on experimentally the data validation and exploration of possible mechanisms of FDX1 in tumorigenesis. Second, we have shown that in the link between FDX1 expression and TMB, MSI lacks any mechanistic explanation from supporting experimental data. More experimental evidence is needed to prove this.

## Conclusion

FDX1 is highly expressed in a variety of tumors, and this high expression is associated with better survival and disease progression, especially for KIRC. FDX1 expression is also associated with immune cell infiltration of tumors, immune checkpoint gene expression, and immunotherapy markers (e.g., TMB and MSI). Taken together, the data suggest that FDX1 provides a valuable new biomarker for several cancers for assessing prognosis and immunotherapy response.

## Data Availability

The datasets presented in this study can be found in online repositories. The names of the repository/repositories and accession number(s) can be found in the article/supplementary material.
